# Malignant External Otitis Complicated by Severe Allergic Reaction to Antibiotic Therapy

**DOI:** 10.7759/cureus.111398

**Published:** 2026-06-23

**Authors:** Jetmira Fejza

**Affiliations:** 1 Otolaryngology, University of Medicine Tirana, Tirana, ALB

**Keywords:** antibiotic allergy, ciprofloxacin, elderly patient, malignant external otitis, necrotizing otitis externa, non-diabetic patient, skull base osteomyelitis, temporal bone osteomyelitis

## Abstract

Malignant external otitis (MEO), also known as necrotizing otitis externa, is an aggressive infection of the external auditory canal and skull base, typically affecting elderly diabetic or immunocompromised patients. We present a case of an 88-year-old non-diabetic woman diagnosed with MEO whose management was complicated by a severe allergic reaction to systemic antibiotic therapy. The patient initially presented with severe otalgia and purulent otorrhea of the right ear, unresponsive to conventional treatment. Otoscopic examination revealed granulation tissue at the osseocartilaginous junction of the external auditory canal. Computed tomography (CT) confirmed temporal bone osteomyelitis consistent with MEO. During hospitalization, the patient developed a severe generalized allergic dermatitis requiring discontinuation of all antibiotics and temporary transfer to the dermatology department. Following stabilization, treatment was resumed with intravenous ciprofloxacin and vancomycin, resulting in progressive clinical improvement and complete recovery after prolonged outpatient therapy. This case highlights the importance of individualized therapeutic strategies in MEO, particularly in elderly patients with atypical presentations and treatment-limiting complications.

## Introduction

Malignant external otitis (MEO) is a severe invasive infection originating in the external auditory canal and extending to the temporal bone and skull base. First described by Meltzer and Kelemen in 1959 and later termed “malignant” by Chandler because of its high mortality rate, the disease remains a potentially life-threatening condition despite advances in antimicrobial therapy [1,2]. The condition predominantly affects elderly diabetic patients and immunocompromised individuals, with *Pseudomonas aeruginosa* being the most commonly isolated pathogen [3]. Clinically, patients usually present with severe persistent otalgia, purulent otorrhea, granulation tissue in the external auditory canal, and, in advanced cases, cranial nerve deficits [4]. Management requires prolonged systemic antipseudomonal therapy, local debridement, and strict control of underlying systemic diseases. However, treatment may become particularly challenging when complications related to antibiotic therapy arise. Although prolonged antipseudomonal antibiotic therapy remains the cornerstone of treatment for malignant external otitis, antibiotic hypersensitivity is rarely reported as a treatment limitation. Such reactions may significantly complicate management by restricting first-line therapeutic options, potentially delaying infection control, and increasing the risk of adverse outcomes. We report a rare case of MEO in an elderly non-diabetic woman complicated by a severe allergic reaction to antibiotic treatment.

## Case presentation

An 88-year-old woman presented to our tertiary ear, nose, and throat (ENT) clinic with severe right-sided otalgia and purulent ear discharge of two weeks’ duration. The patient reported progressive worsening of symptoms despite prior hospitalization in a regional hospital where she had received intravenous ceftriaxone (1 g twice daily) and gentamicin (80 mg twice daily) for five days without improvement of clinical signs. The patient had no history of diabetes mellitus or immunosuppressive disease. Her medical history was significant only for arterial hypertension controlled with medication. On physical examination, the patient appeared in pain but was hemodynamically stable. Otoscopic examination revealed diffuse edema of the external auditory canal, purulent discharge, and granulation tissue located at the osseocartilaginous junction of the canal. Histopathological examination of tissue obtained from the external auditory canal demonstrated granulation tissue, with no evidence of malignancy. To identify a causative pathogen, ear canal swabs were collected on three consecutive days; however, all cultures were negative. Laboratory tests demonstrated elevated erythrocyte sedimentation rate (ESR), while leukocyte count and serum glucose levels were within normal limits. Initial laboratory investigations are summarized in Table 1.

**Table 1:** Initial laboratory investigation results. AST: aspartate aminotransferase; ALT: alanine aminotransferase

Parameters	Patient values	Reference range
White blood cell count (WBC)	7.2×10³/µL	4.0-10.0×10³/µL
Neutrophils	72%	40-75%
Eosinophils	5%	0-6%
Hemoglobin (Hb)	13.2 g/dL	12-16 g/dL
Platelets	286×10³/µL	150-400×10³/µL
C-reactive protein (CRP)	4.2 mg/L	<5 mg/L
Erythrocyte sedimentation rate (ESR)	54 mm/h	<20 mm/h
Blood glucose	96 mg/dL	70-110 mg/dL
HbA1c	5.4%	4.0-5.6%
Serum creatinine	0.9 mg/dL	0.6-1.2 mg/dL
AST	24 U/L	10-40 U/L
ALT	28 U/L	7-56 U/L

Imaging findings

High-resolution CT scan of the temporal bone demonstrated inflammatory soft tissue occupying the right external auditory canal with erosive changes involving the temporal bone, findings compatible with malignant external otitis (Figures 1, 2).

**Figure 1:**
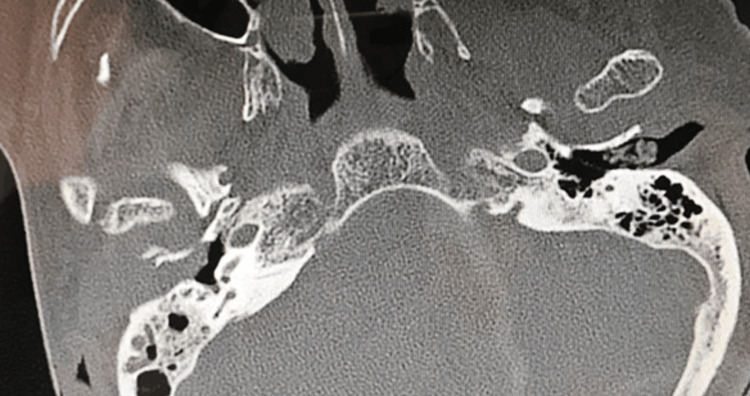
High-resolution CT scan demonstrating inflammatory soft tissue and bony erosion involving the anterior wall of the right external auditory canal.

**Figure 2:**
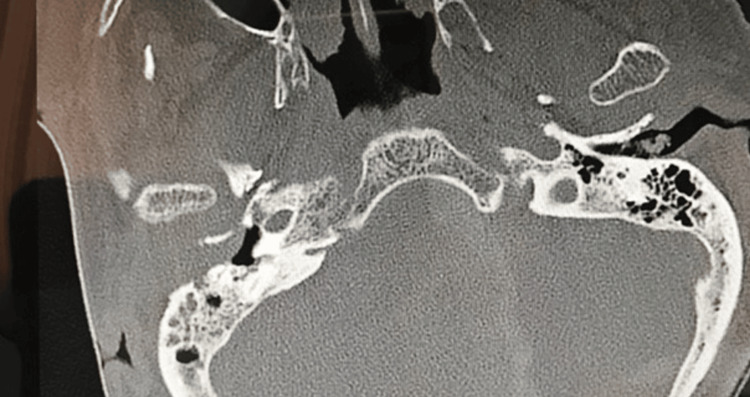
High-resolution CT scan demonstrating bony erosion involving the posterior wall of the right external auditory canal.

Despite the absence of microbiological confirmation, the patient’s clinical deterioration, together with characteristic otoscopic and imaging findings, supported the diagnosis of malignant otitis externa (MOE). Empirical intravenous antimicrobial therapy consisting of levofloxacin 750 mg once daily and cefepime 2 g twice daily was initiated.

Complication during treatment

During hospitalization, on the second day of treatment, the patient developed a severe generalized allergic dermatitis characterized by diffuse erythematous skin eruption and pruritus shortly after antibiotic administration. Cefepime was suspected to be the cause of the hypersensitivity reaction. Due to the severity of the reaction, all systemic antibiotics were discontinued, and the patient was transferred to the dermatology department for management (Figures 3-5).

**Figure 3:**
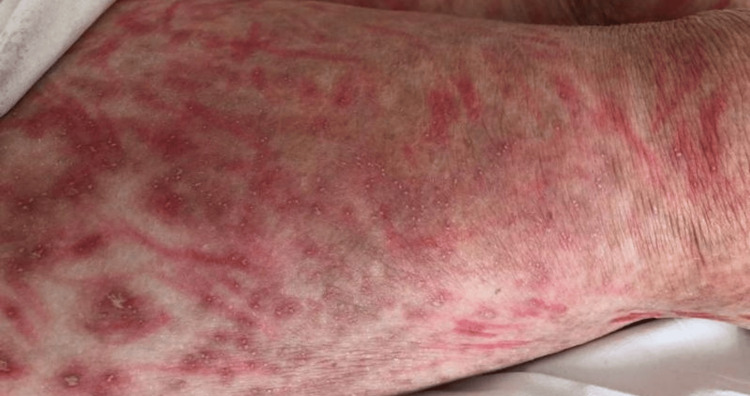
Diffuse erythematous skin eruption following antibiotic therapy (forearm region).

**Figure 4:**
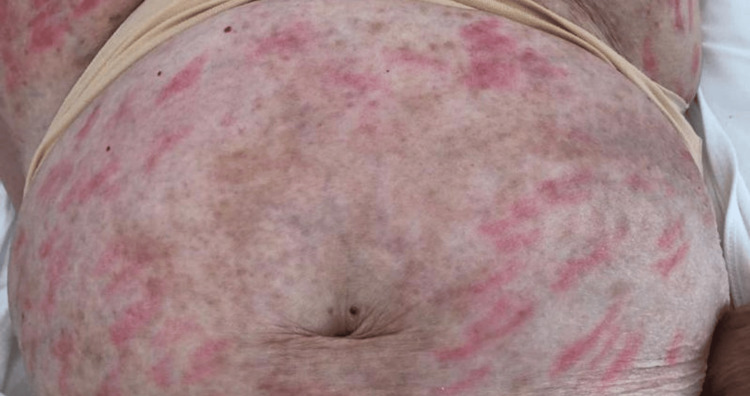
Severe generalized allergic dermatitis during hospitalization (abdominal region).

**Figure 5:**
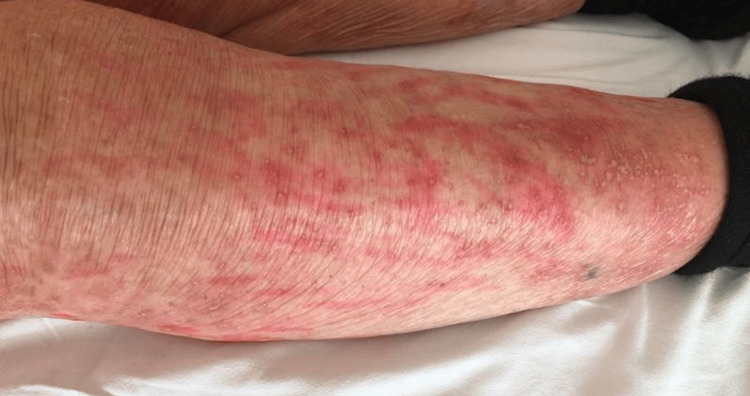
Severe generalized allergic dermatitis (lower extremities).

She received systemic corticosteroids and supportive therapy for one week with gradual clinical improvement of the dermatologic condition. No systemic treatment for the ear condition was administered during the patient's stay in the dermatology department; only local treatment was provided.

Local management included daily microscopic cleaning of the external auditory canal, removal of debris and granulation tissue, and topical quinolone ear drops. Following multidisciplinary consultation with infectious disease specialists and dermatologists, a new antimicrobial regimen consisting of intravenous ciprofloxacin (400 mg twice daily) and vancomycin (1 g twice daily) was initiated cautiously.

Outcome and follow-up

The patient remained hospitalized for approximately two additional weeks after transfer back to the ENT department, resulting in a total hospitalization period of nearly four weeks. At discharge, otalgia had significantly improved, and otorrhea had resolved. Granulation tissue was markedly reduced; no cranial nerve deficits were present. The patient continued outpatient oral ciprofloxacin therapy (750 mg twice daily) for three months with regular ENT follow-up for one year.

The patient was subsequently followed monthly for one year with otologic examinations and erythrocyte sedimentation rate (ESR) monitoring. Clinical findings and inflammatory markers normalized from the second month onward, with complete resolution of symptoms, normal otoscopic findings, and no evidence of disease recurrence. Given the patient’s favorable clinical course and normal findings on follow-up examinations, repeat CT imaging was not considered necessary.

## Discussion

MEO is classically associated with diabetes mellitus and immunosuppression. However, advanced age itself is an important risk factor due to impaired microvascular circulation, immunosenescence, and reduced local tissue resistance [3,5]. The incidence of malignant external otitis appears to be increasing in recent years, likely due to aging populations and the rising prevalence of diabetes mellitus and immunocompromised conditions [6]. Close monitoring of inflammatory parameters is the main factor in determining prognosis [7]. The hallmark symptom of MEO is severe nocturnal otalgia accompanied by otorrhea and granulation tissue at the bone-cartilage junction of the external auditory canal [4,8]. Imaging plays a crucial role in diagnosis. A CT scan is particularly useful for identifying bone erosion and temporal bone osteomyelitis, whereas an MRI is better at evaluating soft-tissue extension and intracranial complications [9]. The present case is notable for several reasons. The patient was non-diabetic despite advanced age. The disease was refractory to initial empirical therapy. Management was complicated by severe allergic reactions to antibiotics. Standard treatment involves prolonged systemic antipseudomonal antibiotics. Fluoroquinolones remain the cornerstone of therapy due to excellent tissue penetration and activity against *Pseudomonas aeruginosa* [10]. In this patient, antibiotic selection became challenging after severe allergic dermatitis developed, necessitating discontinuation of initial therapy and careful reintroduction of alternative agents. The successful outcome illustrates the importance of multidisciplinary collaboration, individualized antimicrobial strategies, long-term follow-up, and careful management of treatment-related complications.

## Conclusions

Malignant external otitis can occur even in non-diabetic elderly patients and should be suspected in cases of persistent otitis externa associated with severe pain and granulation tissue. CT imaging is essential for confirming the diagnosis and assessing the extent of disease. Severe allergic reactions to antibiotics may significantly complicate management and require individualized multidisciplinary therapeutic approaches. Early diagnosis and prolonged targeted treatment remain critical for favorable outcomes.
